# Distinct temporal neuropsychiatric outcomes associated with GLP-1 receptor agonists versus SGLT2 inhibitors after acute kidney disease in type 2 diabetes

**DOI:** 10.3389/fendo.2026.1963944

**Published:** 2026-10-27

**Authors:** Yu-Chen Cheng, Yuahn-Sieh Huang, Jui-Yi Chen, Vin-Cent Wu, Ruei-Ming Chen, Kuo-Cheng Lu, Ko-Lin Kuo, Chien-Lin Lu, Cai-Mei Zheng, Yi-Chou Hou

**Affiliations:** 1 School of Medicine, College of Medicine, Fu Jen Catholic University, New Taipei City, Taiwan; 2 Department of Neurology, Fu Jen Catholic University Hospital, Fu Jen Catholic University, New Taipei City, Taiwan; 3 Department of Biology and Anatomy, National Defense Medical Center, Taipei, Taiwan; 4 Division of Nephrology, Department of Internal Medicine, Chi Mei Medical Center, Tainan, Taiwan; 5 Department of Health and Nutrition, Chia Nan University of Pharmacy and Science, Tainan, Taiwan; 6 Department of Public Health, College of Medicine, National Cheng Kung University, Tainan, Taiwan; 7 Division of Nephrology, Department of Internal Medicine, National Taiwan University Hospital, Taipei, Taiwan; 8 NSARF (National Taiwan University Hospital Study Group of Acute Renal Failure) and Taiwan Consortium for Acute Kidney Injury and Renal Diseases (CAKS), Taipei, Taiwan; 9 Cell Physiology and Molecular Image Research Center, Wan Fang Hospital, Taipei Medical University, Taipei, Taiwan; 10 Graduate Institute of Medical Sciences, College of Medicine, Taipei Medical University, Taipei, Taiwan; 11 Department of Anesthesiology and Health Policy Research Center, Taipei Medical University Hospital, Taipei Medical University, Taipei, Taiwan; 12 Division of Nephrology, Department of Medicine, Taipei Tzu Chi Hospital, Buddhist Tzu Chi Medical Foundation, New Taipei City, Taiwan; 13 School of Medicine, Tzu Chi University, Hualien, Taiwan; 14 School of Post-Baccalaureate Chinese Medicine, Tzu Chi University, Hualien, Taiwan; 15 Division of Nephrology, Department of Internal Medicine, Fu Jen Catholic University Hospital, Fu Jen Catholic University, New Taipei City, Taiwan; 16 Division of Nephrology, Department of Internal Medicine, Shuang Ho Hospital, Taipei Medical University, New Taipei City, Taiwan; 17 TMU Research Center of Urology and Kidney, Taipei Medical University, Taipei, Taiwan; 18 Division of Nephrology, Department of Internal Medicine, Cardinal Tien Hospital, New Taipei City, Taiwan

**Keywords:** acute kidney disease, delirium, depression, GLP-1 RA, SGLT2 (sodium-glucose cotransporter 2) inhibitor, type 2 diabetes

## Abstract

**Background:**

Neurological and neuropsychiatric complications may persist after acute kidney disease (AKD), particularly among patients with type 2 diabetes mellitus (T2DM). Although glucagon-like peptide-1 receptor agonists (GLP-1RAs) and sodium-glucose cotransporter-2 inhibitors (SGLT2is) provide established cardiorenal benefits, their associations with neurological outcomes after AKD remain uncertain.

**Methods:**

We conducted a retrospective cohort study using the TriNetX Global Collaborative Network. Adults with T2DM and dialysis-requiring AKD receiving GLP-1RAs or SGLT2is were identified and balanced using 1:1 propensity score matching, yielding 3,398 patients in each group. Incident delirium and other neuropsychiatric outcomes were evaluated. The 90–365-day period was the primary analysis, with 90–180- and 90–720-day windows used to assess temporal patterns. Kaplan–Meier and multivariable Cox regression analyses were performed.

**Results:**

During 90–365 days, GLP-1RA use was associated with better delirium-free survival than SGLT2i use (118/3,032 vs. 155/2,982 events; log-rank *P* = 0.009). This association remained significant after multivariable adjustment (adjusted hazard ratio [HR], 0.668; 95% CI, 0.488–0.914; P = 0.012). Statin use was independently associated with lower delirium risk (adjusted HR, 0.674; 95% CI, 0.492–0.924; P = 0.014), while exploratory analyses suggested a distinct association between LDL cholesterol of 100–129.9 mg/dL and delirium risk. In contrast, SGLT2i use showed a favorable association with depression during early follow-up, which attenuated over time. The GLP-1RA–delirium association was also attenuated by 90–720 days. Exploratory sensitivity analyses using non-overlapping follow-up intervals showed outcome-specific associations: adjusted differences in delirium were not significant, whereas depression and overall neurological complications were more frequent with GLP-1RA than with SGLT2i.

**Conclusions:**

Among patients with dialysis-requiring AKD and T2DM, GLP-1RA and SGLT2i use showed outcome-specific associations with subsequent neuropsychiatric events. Although the primary analysis suggested a lower delirium risk with GLP-1RA, this association was not consistently observed in adjusted time-stratified analyses. These findings should be considered exploratory and require prospective validation.

## Introduction

Acute kidney injury (AKI) is common and clinically important in patients with type 2 diabetes mellitus (T2DM), in whom metabolic, microvascular, cardiovascular, and kidney abnormalities increase susceptibility to acute and persistent kidney dysfunction (1). Although AKI has traditionally been viewed as an acute event, acute kidney disease (AKD) represents persistent kidney dysfunction after AKI and bridges AKI and chronic kidney disease (CKD) (2). Persistent or severe kidney injury is associated with long-term risks of CKD progression, cardiovascular events, and mortality (3). These risks are particularly relevant in diabetes, where recurrent kidney injury and cardiorenal-metabolic abnormalities may compromise recovery. Diabetic AKD therefore represents a distinct therapeutic challenge (4). Emerging real-world evidence suggests that contemporary glucose-lowering therapies may modify post-AKD outcomes. SGLT2 inhibitor use after AKD has been associated with favorable kidney, cardiovascular, and survival outcomes in patients with T2DM (5), highlighting pharmacological strategies beyond glycemic control.

Beyond kidney and cardiovascular outcomes, neurological and neuropsychiatric complications are increasingly recognized as post-AKI and post-critical illness morbidity (6). AKI is linked to acute and long-term neurological consequences through systemic and neuroinflammation, endothelial dysfunction, blood–brain barrier disruption, oxidative stress, uremic toxin accumulation, and altered cerebral metabolism (6). Delirium is especially relevant because systemic inflammation may trigger endothelial and blood–brain barrier injury, microglial activation, oxidative stress, and disruption of neuronal networks (7), while delirium during critical illness is associated with later cognitive impairment (8). Biomarkers of endothelial activation and blood–brain barrier injury have also been associated with delirium in critically ill patients, supporting a kidney–systemic–brain interaction during and after severe illness (9). Depression, cognitive impairment, and dementia may further impair recovery and quality of life. However, most AKI and AKD studies have focused on mortality, kidney progression, and cardiovascular events, leaving post-AKD neuropsychiatric outcomes understudied, particularly in diabetes.

No established pharmacological strategy specifically prevents neuropsychiatric complications after AKD. However, the expanding cardiorenal indications of glucagon-like peptide-1 receptor agonists (GLP-1RAs) and sodium-glucose cotransporter-2 inhibitors (SGLT2is) raise the possibility of benefits beyond metabolic and cardiorenal outcomes. SGLT2 inhibitors have demonstrated kidney and cardiovascular benefits and favorable post-AKD outcomes in patients with T2DM (5), while comparative evidence suggests differential kidney outcomes between SGLT2 inhibitors and GLP-1RAs in patients with T2DM (10). Accumulating experimental and clinical evidence also suggests that GLP-1 receptor activation may affect neuroinflammation, microglial activity, oxidative stress, mitochondrial function, and cerebral glucose metabolism, supporting biological plausibility for neurological effects. Whether these two glucose-lowering drug classes are associated with different post-AKD neuropsychiatric outcomes remains unclear. Therefore, using a large multinational real-world database, we compared incident delirium, depression, and other neurological outcomes between GLP-1RA and SGLT2 inhibitor users with T2DM after dialysis-requiring AKD. We also examined whether these associations differed across early, intermediate, and extended follow-up to explore outcome-specific and temporal patterns of neurological vulnerability during post-AKD recovery.

## Materials and Methods

### Study design and data source

We conducted a retrospective, multinational cohort study using the TriNetX Global Collaborative Network, a federated electronic health record (EHR) platform with de-identified longitudinal clinical data from participating healthcare organizations (HCOs). The database includes demographics, diagnoses, procedures, medications, laboratory measurements, and healthcare encounters. The query was generated on May 16, 2026; 173 HCOs responded, and 45 contributed eligible patients. Source data spanned September 1, 2012, to September 30, 2023.

All analyses were performed within the secure TriNetX analytics environment using de-identified data. Diagnoses were identified primarily using International Classification of Diseases, Tenth Revision, Clinical Modification (ICD-10-CM) codes; medications using Anatomical Therapeutic Chemical (ATC) codes; procedures using Current Procedural Terminology (CPT), Healthcare Common Procedure Coding System (HCPCS), SNOMED CT, and relevant ICD procedure codes; and laboratory measurements using Logical Observation Identifiers Names and Codes (LOINC).

### Study population and definition of acute kidney disease

We identified adults aged 18–80 years with type 2 diabetes mellitus (T2DM) who had a qualifying hospitalization with severe kidney dysfunction requiring dialysis or renal replacement therapy. Hospital discharge was identified using CPT codes 99239, 99238, and 99217 with corresponding grouped discharge concepts. To establish temporality, dialysis or renal replacement therapy was required from 3 months before to 1 day after discharge.

In this study, AKD was defined using a prespecified EHR-based hospitalization–dialysis phenotype rather than retrospective reconstruction of Kidney Disease: Improving Global Outcomes (KDIGO) serum creatinine criteria. This definition was selected to identify patients with clinically severe, persistent kidney dysfunction within a multinational federated EHR database.

Patients with documented delirium or altered consciousness during the qualifying hospitalization were excluded to reduce misclassification of prevalent or hospitalization-associated delirium as incident post-AKD events. The pre-index exclusion was defined using ICD-10-CM codes F05, R41.82, R41.0, and R40.0. For each outcome analysis, patients with the corresponding outcome documented before the assessment window were also excluded to focus on incident events.

### Exposure definition

Eligible patients were classified into two mutually exclusive cohorts by exposure to contemporary glucose-lowering therapies. The GLP-1RA cohort included patients exposed to a glucagon-like peptide-1 receptor agonist, identified by ATC code A10BJ, without concomitant SGLT2 inhibitor exposure. The SGLT2i cohort included patients exposed to a sodium-glucose cotransporter-2 inhibitor, identified by ATC code A10BK, without GLP-1RA exposure.

The mutually exclusive exposure definition minimized treatment overlap and enabled active-comparator analysis between drug classes. Before propensity score matching, 3,822 patients were identified in the GLP-1RA cohort and 5,937 in the SGLT2i cohort. After 1:1 propensity score matching, 3,398 patients remained in each cohort.

### Baseline characteristics and covariates

Baseline characteristics were assessed before matching and included demographic, metabolic, cardiovascular, kidney, laboratory, and medication variables. Demographics included age and sex. Comorbidities included hypertension, ischemic heart disease, heart failure, cerebrovascular disease, chronic obstructive pulmonary disease, and hyperlipidemia.

Kidney and metabolic variables included estimated glomerular filtration rate (eGFR), urinary albumin-to-creatinine ratio (UACR), body mass index (BMI), and glycated hemoglobin (HbA1c). Laboratory variables included hemoglobin, serum albumin, potassium, C-reactive protein, total cholesterol, low-density lipoprotein cholesterol (LDL-C), high-density lipoprotein cholesterol, and triglycerides, where available.

Medication covariates included cardiovascular and metabolic therapies, including statins, beta-blockers, renin–angiotensin system-modifying therapies, and diuretics. Variables were selected for potential associations with treatment selection, cardiorenal status, metabolic severity, or subsequent neurological outcomes.

Detailed diagnostic, medication, laboratory, and procedural codes used to construct cohorts and covariates are provided in the **Supplementary Table**.

## Propensity score matching

To reduce baseline differences between cohorts, 1:1 propensity score matching (PSM) was performed using demographics, major comorbidities, kidney and metabolic parameters, laboratory measurements, and concomitant medications available in TriNetX.

Covariate balance was assessed using standardized mean differences (SMDs) before and after matching, with smaller absolute SMDs indicating better balance. PSM improved overall comparability, although residual imbalance remained for selected clinically relevant characteristics. Because GLP-1RAs and SGLT2 inhibitors have different guideline-supported indications by metabolic, cardiovascular, and kidney phenotypes, residual confounding by indication was considered when interpreting findings.

### Outcome assessment

The primary objective was to compare incident neurological and neuropsychiatric outcomes between GLP-1RA and SGLT2i users after dialysis-requiring AKD. The primary assessment period was 90–365 days after the index event. The outcomes of interest included incident delirium, depression, and a composite neurological outcome. Incident delirium was identified using the complete prespecified TriNetX outcome concept set, which included multiple diagnosis, procedure, and medication concepts related to delirium and was therefore not restricted to ICD-10-CM F05 alone. Depression and the composite neurological outcome were similarly identified using their prespecified TriNetX concept sets. The complete coding definitions for all outcomes are provided in **Supplementary Table S1**. To minimize contamination by prevalent disease, patients with the corresponding outcome documented before each assessment window were excluded from that outcome-specific analysis. Because neurological manifestations after severe systemic illness may evolve over time, the prespecified analyses additionally examined an early post-AKD window (90–180 days) and an extended follow-up window (90–730 days), in addition to the primary 90–365-day assessment. The 90-day landmark was used to focus on post-acute neurological events rather than complications occurring during hospitalization or early recovery. To further evaluate the robustness of the findings and avoid overlap between follow-up windows, exploratory sensitivity analyses were subsequently performed using three mutually exclusive intervals: 90–180, 181–365, and 366–730 days. Crude Kaplan–Meier analyses and multivariable Cox models using a consistent covariate set were performed for delirium, depression, and the composite neurological outcome across these intervals. These interval-specific analyses were considered exploratory and hypothesis-generating rather than formal tests of treatment-by-time interaction. These prespecified windows assessed whether associations between treatment class and neurological outcomes differed by post-AKD recovery stage.

### Subgroup and exploratory analyses

To characterize factors associated with incident delirium during the primary 90–365-day period, additional analyses examined demographics, kidney function, metabolic and laboratory parameters, and concomitant medication use. Lipid parameters and statin exposure were emphasized because of their potential links with vascular, inflammatory, and neurocognitive outcomes. LDL-C was evaluated using clinically defined ranges, including <100, 100–129.9, and ≥130 mg/dL. These ranges were evaluated as separate indicator variables (presence versus absence of the specified range) rather than as mutually exclusive categorical levels referenced to a single LDL-C category. These analyses were exploratory and were not intended to establish an optimal LDL-C treatment target.

### Statistical analysis

Baseline characteristics were compared between cohorts before and after propensity score matching. Continuous variables were summarized as means with standard deviations, and categorical variables as numbers and percentages. Between-group differences were assessed using appropriate statistical tests within TriNetX, and covariate balance was evaluated using SMDs.

Time-to-event analyses were performed for incident neurological outcomes. Kaplan–Meier methods estimated cumulative event-free probabilities, and log-rank tests compared treatment cohorts. Hazard ratios (HRs) with 95% confidence intervals (CIs) were estimated using Cox proportional hazards models. Death was treated as a censoring event in the primary time-to-event analyses; mortality was additionally compared between treatment groups as a sensitivity assessment for potential differential competing risk.

For incident delirium during the primary 90–365-day period, multivariable Cox proportional hazards regression assessed whether the association between treatment class and delirium persisted after adjustment for clinically relevant demographic, kidney, metabolic, laboratory, and medication covariates. Because residual imbalance remained after propensity score matching for ischemic heart disease, beta-blocker use, and diuretic use, additional multivariable Cox sensitivity analyses were performed. These models consistently adjusted for treatment group, age, sex, ischemic heart disease, beta-blocker use, and diuretic use across all three neuropsychiatric outcomes and the prespecified follow-up intervals.

The 90–365-day interval served as the primary analytical window, whereas 90–180- and 90–730-day analyses characterized early and extended temporal patterns. Because statistical significance across separate windows does not constitute a formal treatment-by-time interaction, differences were interpreted as exploratory temporal patterns rather than definitive evidence of time-varying treatment effects. To facilitate interpretation of temporal patterns, additional exploratory analyses were performed using three non-overlapping follow-up intervals (90–180, 181–365, and 366–730 days). These analyses were conducted separately and were not equivalent to a formal treatment-by-time interaction test; therefore, comparisons of statistical significance across intervals were considered descriptive and hypothesis-generating. Given the multiple secondary outcomes, follow-up intervals, and subgroup analyses, these analyses were considered exploratory and hypothesis-generating. All statistical tests were two-sided, and P<0.05 was considered statistically significant. Analyses were conducted within TriNetX.

## Results

### Baseline characteristics of the study population

After applying the predefined eligibility criteria, 3,822 patients with acute kidney disease (AKD) receiving GLP-1 receptor agonists (GLP-1RAs) without SGLT2 inhibitors (SGLT2is) and 5,937 patients receiving SGLT2is without GLP-1RAs were included in the final study cohorts. After 1:1 propensity score matching, 3,398 patients remained in each treatment group (**Figure 1**).

**Figure 1:**
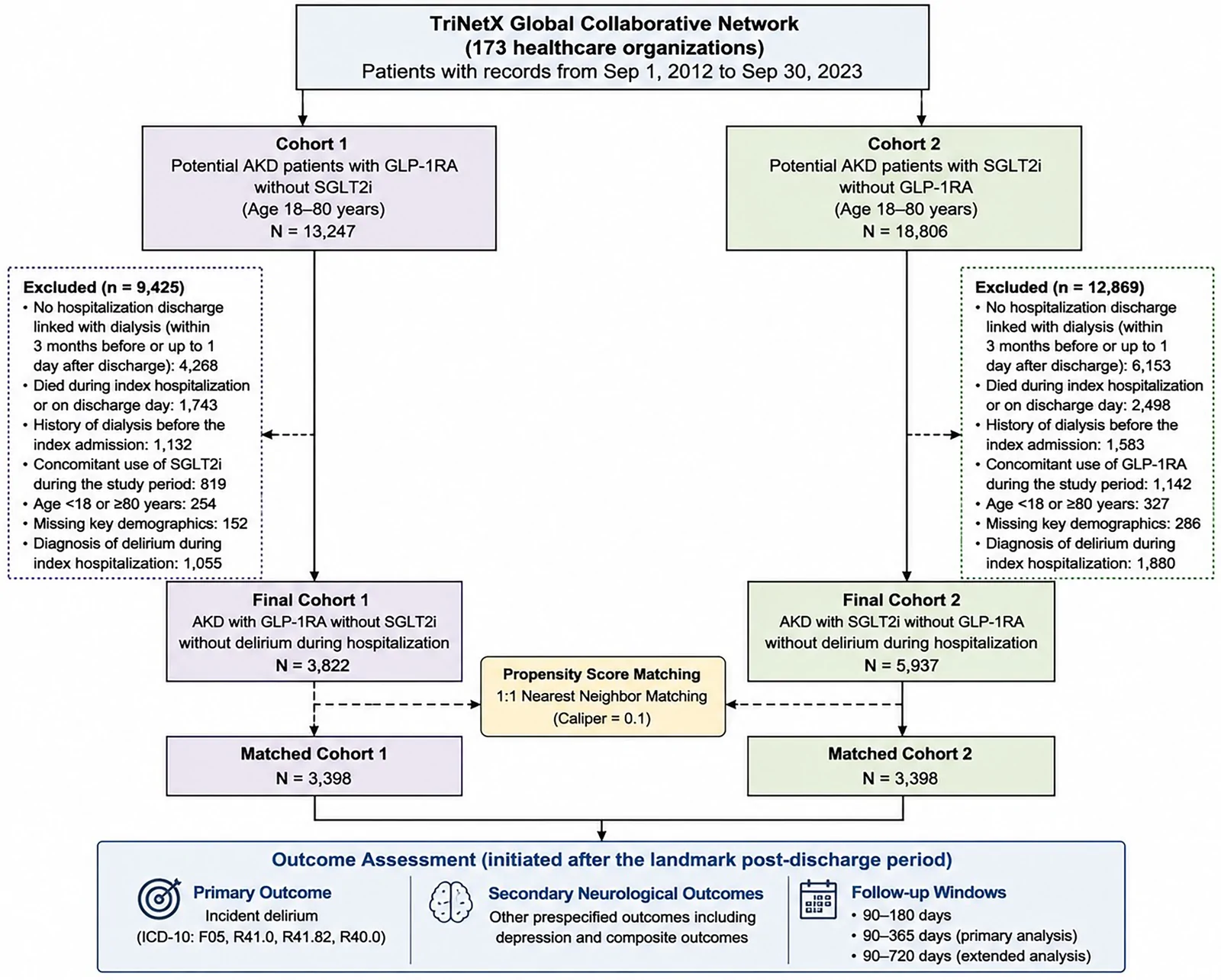
Study cohort selection and analytical framework. Flow diagram illustrating cohort construction from the TriNetX Global Collaborative Network. Patients aged 18–80 years with type 2 diabetes and dialysis-requiring acute kidney disease (AKD) were classified according to exposure to either a glucagon-like peptide-1 receptor agonist (GLP-1RA) without concomitant sodium–glucose cotransporter-2 inhibitor (SGLT2i) use or an SGLT2i without concomitant GLP-1RA use. After applying prespecified exclusion criteria, 3,822 patients in the GLP-1RA cohort and 5,937 patients in the SGLT2i cohort were included before propensity score matching, consistent with the baseline population reported in **Table 1**. One-to-one nearest-neighbor propensity score matching with a caliper of 0.1 yielded 3,398 patients in each group. Incident delirium was the primary outcome, with depression and composite neurological outcomes assessed as secondary outcomes. Outcomes were evaluated during 90–180-, 90–365-, and 90–730-day post-discharge windows, with 90–365 days designated as the primary analysis. AKD, acute kidney disease; GLP-1RA, glucagon-like peptide-1 receptor agonist; SGLT2i, sodium–glucose cotransporter-2 inhibitor.

Baseline demographic, clinical, laboratory, and medication characteristics before and after propensity score matching are summarized in **Table 1**. Before matching, substantial differences were observed between the two treatment groups. Patients receiving GLP-1RAs were younger (55.5 ± 12.2 vs. 59.5 ± 10.7 years, *P* < 0.001; SMD = 0.354), less frequently male (47.6% vs. 62.7%, *P* < 0.001; SMD = 0.308), and had a higher BMI (36.5 ± 9.5 vs. 33.3 ± 8.4 kg/m², *P* < 0.001; SMD = 0.359). Conversely, ischemic heart disease, COPD, and use of several cardiovascular medications were more frequent among SGLT2i users. In particular, ischemic heart disease was present in 30.1% of GLP-1RA users versus 52.2% of SGLT2i users (SMD = 0.461), while beta-blocker use was 50.7% versus 68.6% (SMD = 0.370), respectively.

**Table 1:** Baseline demographic, clinical, laboratory, and medication characteristics of patients receiving GLP-1 receptor agonists or SGLT2 inhibitors before and after propensity score matching.

Characteristics	GLP-1RA Before (n=3,822)	SGLT2i Before (n=5,937)	P value	SMD	GLP-1RA After (n=3,398)	SGLT2i After (n=3,398)	P value	SMD
Demographics								
Age at index, years	55.5 ± 12.2	59.5 ± 10.7	<0.001	0.354	57.2 ± 11.1	57.1 ± 11.2	0.803	0.006
Male sex, n (%)	1,817 (47.6%)	3,701 (62.7%)	<0.001	0.308	1,750 (51.5%)	1,750 (51.5%)	1.000	<0.001
Comorbidities								
Overweight/obesity, n (%)	2,185 (57.2%)	2,774 (47.0%)	<0.001	0.205	1,872 (55.1%)	1,766 (52.0%)	0.010	0.063
Hypertension, n (%)	3,216 (84.2%)	5,270 (89.3%)	<0.001	0.152	2,952 (86.9%)	2,965 (87.3%)	0.638	0.011
Ischemic heart disease, n (%)	1,148 (30.1%)	3,078 (52.2%)	<0.001	0.461	1,087 (32.0%)	1,574 (46.3%)	<0.001	0.297
Cerebrovascular disease, n (%)	388 (10.2%)	775 (13.1%)	<0.001	0.093	373 (11.0%)	395 (11.6%)	0.399	0.020
COPD, n (%)	522 (13.7%)	1,135 (19.2%)	<0.001	0.151	486 (14.3%)	617 (18.2%)	<0.001	0.105
Hyperlipidemia, n (%)	2,265 (59.3%)	3,694 (62.6%)	0.001	0.068	2,075 (61.1%)	2,041 (60.1%)	0.399	0.020
Kidney and metabolic characteristics								
BMI, kg/m²	36.5 ± 9.5	33.3 ± 8.4	<0.001	0.359	35.9 ± 9.3	35.0 ± 8.8	0.001	0.095
eGFR, mL/min/1.73 m²	81.2 ± 31.7	78.4 ± 27.8	<0.001	0.094	80.2 ± 30.7	80.4 ± 29.6	0.782	0.007
UACR, mg/g	757.4 ± 2697.2	801.0 ± 3105.4	0.844	0.015	828.7 ± 2972.2	864.8 ± 3552.3	0.901	0.011
Laboratory parameters								
Hemoglobin, g/dL	11.7 ± 2.1	12.0 ± 2.4	<0.001	0.127	11.8 ± 2.1	12.0 ± 2.3	<0.001	0.089
Total cholesterol, mg/dL	167.1 ± 68.0	157.7 ± 72.2	<0.001	0.134	164.8 ± 66.0	164.5 ± 77.5	0.925	0.003
LDL cholesterol, mg/dL	87.8 ± 42.6	83.2 ± 41.6	<0.001	0.110	86.3 ± 42.4	86.6 ± 43.5	0.843	0.007
HDL cholesterol, mg/dL	37.4 ± 14.9	35.2 ± 16.6	<0.001	0.139	37.6 ± 15.1	36.5 ± 15.7	0.052	0.069
Triglycerides, mg/dL	222.6 ± 256.0	184.6 ± 244.0	<0.001	0.152	216.6 ± 251.0	204.2 ± 283.6	0.182	0.046
HbA1c, %	9.1 ± 2.7	8.4 ± 2.5	<0.001	0.255	9.0 ± 2.7	8.6 ± 2.5	<0.001	0.139
Potassium, mmol/L	4.0 ± 0.5	4.0 ± 0.5	0.051	0.041	4.0 ± 0.5	4.0 ± 0.5	0.618	0.012
Medications								
DPP-4 inhibitors, n (%)	250 (6.5%)	414 (7.0%)	0.368	0.019	229 (6.7%)	252 (7.4%)	0.277	0.026
Biguanides, n (%)	1,305 (34.2%)	1,720 (29.2%)	<0.001	0.108	1,133 (33.3%)	1,120 (33.0%)	0.738	0.008
RAAS inhibitors, n (%)	2,065 (54.1%)	3,784 (64.1%)	<0.001	0.206	1,958 (57.6%)	1,972 (58.0%)	0.731	0.008
Beta-blockers, n (%)	1,937 (50.7%)	4,045 (68.6%)	<0.001	0.370	1,787 (52.6%)	2,160 (63.6%)	<0.001	0.224
Diuretics, n (%)	1,916 (50.2%)	3,918 (66.4%)	<0.001	0.334	1,748 (51.4%)	2,124 (62.5%)	<0.001	0.225
Calcium-channel blockers, n (%)	1,321 (34.6%)	2,404 (40.8%)	<0.001	0.127	1,224 (36.0%)	1,276 (37.6%)	0.191	0.032
Statins, n (%)	2,384 (62.4%)	4,280 (72.6%)	<0.001	0.218	2,199 (64.7%)	2,350 (69.2%)	<0.001	0.095
Benzodiazepine derivatives, n (%)	127 (3.3%)	160 (2.7%)	0.081	0.036	113 (3.3%)	101 (3.0%)	0.405	0.020
Antipsychotics, n (%)	817 (21.4%)	1,129 (19.1%)	0.007	0.056	701 (20.6%)	700 (20.6%)	0.976	0.001

Baseline characteristics are presented for the GLP-1RA and SGLT2i cohorts before and after 1:1 propensity score matching. Continuous variables are reported as mean ± standard deviation and categorical variables as number (%). P values represent between-group comparisons. Standardized mean differences (SMDs) are provided to describe covariate balance.

GLP-1RA, glucagon-like peptide-1 receptor agonist; SGLT2i, sodium-glucose cotransporter-2 inhibitor; SMD, standardized mean difference; COPD, chronic obstructive pulmonary disease; BMI, body mass index; eGFR, estimated glomerular filtration rate; UACR, urine albumin-to-creatinine ratio; LDL, low-density lipoprotein; HDL, high-density lipoprotein; HbA1c, glycated hemoglobin A1c; DPP-4, dipeptidyl peptidase-4; RAAS, renin-angiotensin-aldosterone system.

Propensity score matching substantially improved overall covariate balance. Age, sex, hypertension, cerebrovascular disease, hyperlipidemia, kidney function, UACR, lipid parameters, and several medication exposures were well balanced after matching. Nevertheless, residual differences persisted for selected characteristics, particularly ischemic heart disease (32.0% vs. 46.3%; SMD = 0.297), COPD (14.3% vs. 18.2%; SMD = 0.105), HbA1c (9.0 ± 2.7% vs. 8.6 ± 2.5%; SMD = 0.139), beta-blocker use (52.6% vs. 63.6%; SMD = 0.224), and diuretic use (51.4% vs. 62.5%; SMD = 0.225). Given these residual imbalances, ischemic heart disease, beta-blocker use, and diuretic use were included, together with age and sex, in additional multivariable Cox sensitivity analyses. BMI showed a smaller residual difference after matching (35.9 ± 9.3 vs. 35.0 ± 8.8 kg/m²; SMD = 0.095). These remaining imbalances were considered in subsequent multivariable analyses.

### Primary neuropsychiatric outcomes during 90–365 days

The prespecified primary analysis evaluated neurological and neuropsychiatric outcomes between 90 and 365 days after the index hospitalization (**Figure 2**). The most prominent between-group difference was observed for incident delirium. Patients receiving GLP-1RAs had a significantly lower risk of delirium than those receiving SGLT2is, with an HR of 0.57 (95% CI, 0.39–0.82; *P* = 0.002). The separation between the two curves became increasingly apparent during follow-up, suggesting that the relative difference in delirium risk emerged during the post-acute recovery period rather than immediately after discharge.

**Figure 2:**
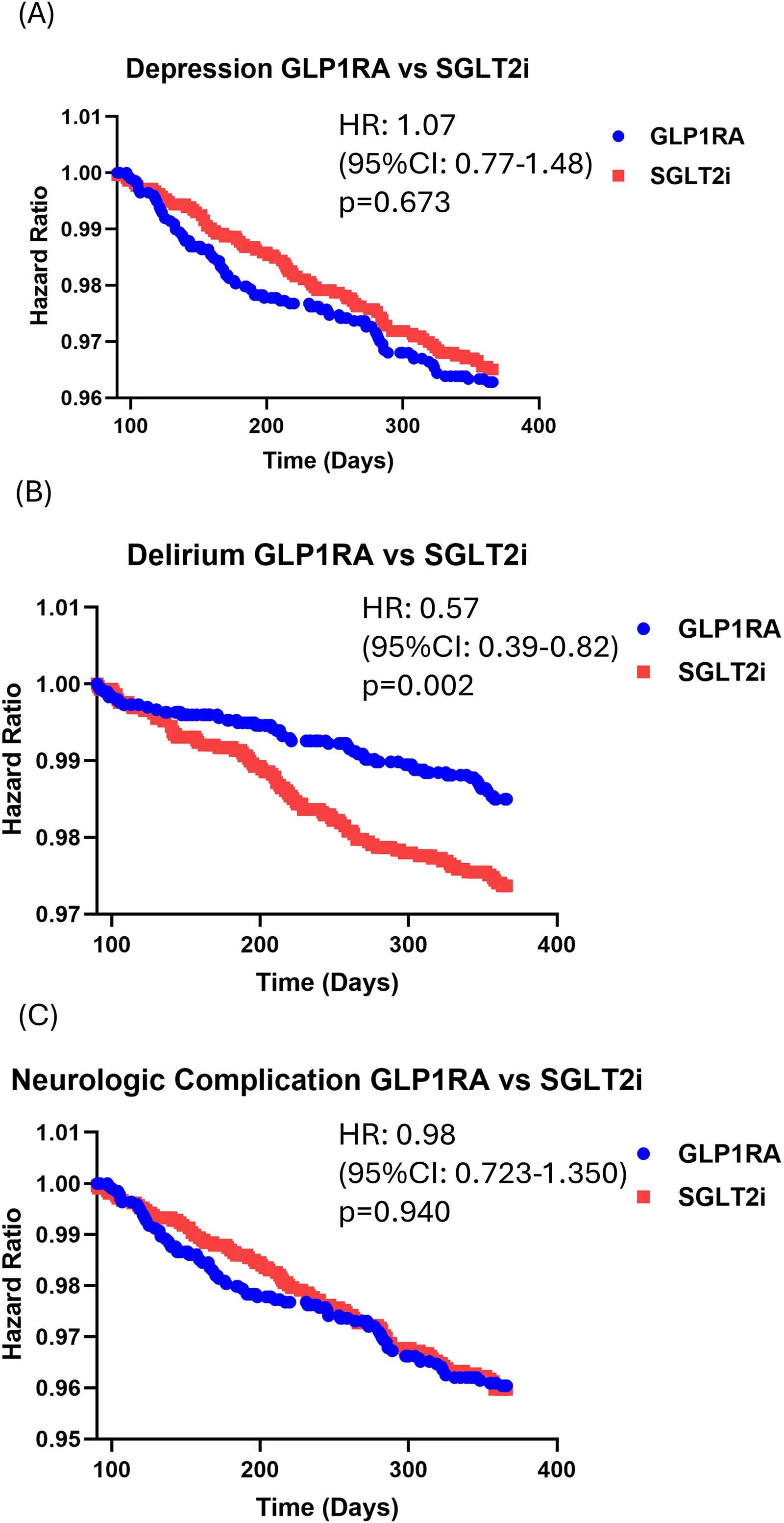
Neurological outcomes associated with GLP-1RA versus SGLT2i use during the 90–365-day follow-up period. Time-to-event analyses comparing GLP-1RA and SGLT2i users for **(A)** depression, **(B)** incident delirium, and **(C)** composite neurological complications during the primary 90–365-day analysis window. GLP-1RA use was associated with a significantly lower risk of incident delirium compared with SGLT2i use (HR, 0.57; 95% CI, 0.39–0.82; *P* = 0.002), whereas no significant between-group differences were observed for depression (HR, 1.07; 95% CI, 0.77–1.48; *P* = 0.673) or composite neurological complications (HR, 0.98; 95% CI, 0.723–1.350; *P* = 0.940). CI, confidence interval; GLP-1RA, glucagon-like peptide-1 receptor agonist; HR, hazard ratio; SGLT2i, sodium–glucose cotransporter-2 inhibitor.

In contrast, no significant difference was observed for depression during the 90–365-day primary window (HR, 1.07; 95% CI, 0.77–1.48; *P* = 0.673). Similarly, the composite neurological outcome was comparable between the two groups (HR, 0.98; 95% CI, 0.723–1.350; *P* = 0.940). Thus, the neurological association observed during the primary follow-up period appeared to be outcome-specific, predominantly driven by a lower incidence of delirium in the GLP-1RA group, rather than a generalized reduction in all neurological or neuropsychiatric complications.

In additional analyses using non-overlapping follow-up intervals, crude Kaplan–Meier estimates showed that the between-group difference in depression was significant during 90–180 days (HR, 1.572; 95% CI, 1.003–2.463; P = 0.0465), but not during 181–365 or 366–730 days. A similar early difference was observed for overall neurological complications during 90–180 days (HR, 1.559; 95% CI, 1.001–2.428; P = 0.0478), whereas no significant between-group difference in delirium was observed across any of the three intervals. Event counts and complete crude estimates are provided in **Supplementary Table 2**. After additional adjustment for age, sex, ischemic heart disease, diuretic use, and beta-blocker use, GLP-1RA treatment was associated with a higher hazard of depression and overall neurological complications across all three non-overlapping intervals. In contrast, the adjusted association with delirium remained nonsignificant during 90–180 days (HR, 0.778; 95% CI, 0.528–1.148; P = 0.2067), 181–365 days (HR, 0.814; 95% CI, 0.609–1.089; P = 0.1660), and 366–730 days (HR, 0.926; 95% CI, 0.735–1.168; P = 0.5180). Complete adjusted estimates are provided in **Supplementary Table 3**. In an additional agent-specific sensitivity analysis, empagliflozin and semaglutide were the most frequently represented individual agents, accounting for approximately 83.7% (4,971/5,937) of the SGLT2i cohort and 53.3% (2,039/3,822) of the GLP-1RA cohort, respectively. After 1:1 propensity score matching (n=1,951 per group), no significant differences were observed for depression (HR 0.880, 95% CI 0.575–1.347; P = 0.557), delirium (HR 1.556, 95% CI 0.920–2.634; P = 0.097), or overall neurological complications (HR 1.091, 95% CI 0.735–1.620; P = 0.665) (**Supplementary Table S4**).

### Factors associated with incident delirium during the primary follow-up period

To determine whether the observed association with delirium was independent of baseline clinical characteristics, laboratory parameters, and concomitant medications, multivariable Cox proportional hazards analyses were performed for the 90–365-day period (**Figure 3**).

**Figure 3:**
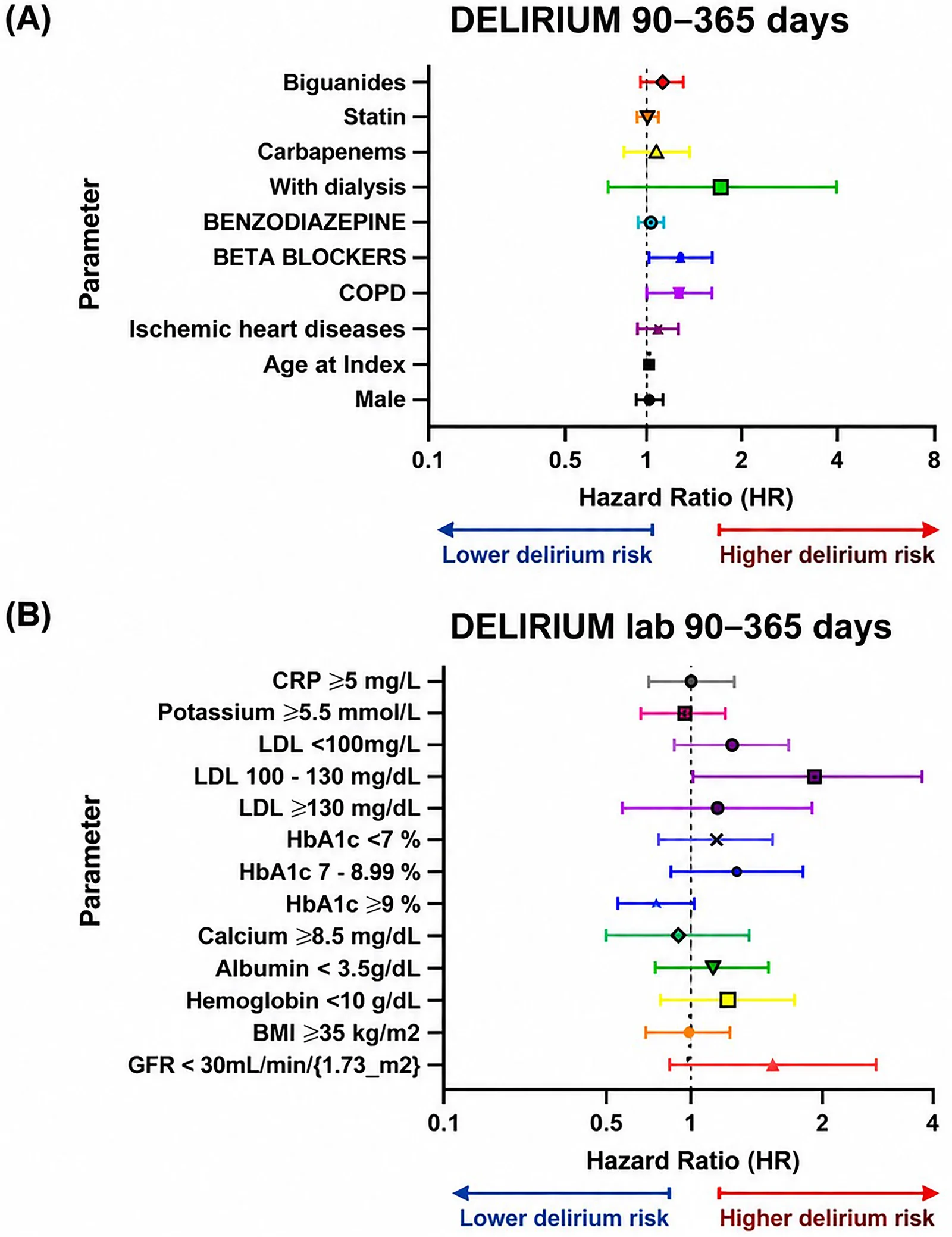
Multivariable Cox proportional hazards analyses of factors associated with incident delirium during the 90–365-day follow-up period. **(A)** Clinical characteristics and medication-related factors associated with incident delirium. **(B)** Laboratory and metabolic factors associated with incident delirium. Hazard ratios (HRs) with 95% confidence intervals (CIs) are presented. An HR <1 indicates an association with a lower hazard of incident delirium, whereas an HR >1 indicates an association with a higher hazard. The vertical dashed line represents HR = 1. These covariate-specific HRs represent associations with incident delirium and should not be interpreted as direct comparisons between GLP-1RA and SGLT2i treatment groups. BMI, body mass index; CI, confidence interval; COPD, chronic obstructive pulmonary disease; CRP, C-reactive protein; GFR, glomerular filtration rate; GLP-1RA, glucagon-like peptide-1 receptor agonist; HbA1c, glycated hemoglobin A1c; HR, hazard ratio; LDL, low-density lipoprotein; SGLT2i, sodium–glucose cotransporter-2 inhibitor.

After multivariable adjustment, GLP-1RA use remained independently associated with a lower risk of incident delirium compared with SGLT2i use (adjusted HR, 0.668; 95% CI, 0.488–0.914; *P* = 0.012). Importantly, statin use was also independently associated with a lower delirium risk (adjusted HR, 0.674; 95% CI, 0.492–0.924; *P* = 0.014).

Among laboratory parameters, LDL cholesterol of 100–129.9 mg/dL showed an association with a higher hazard of incident delirium. LDL-C ranges were evaluated as separate indicator variables rather than as mutually exclusive categories referenced to a single LDL-C stratum. In contrast, most other evaluated clinical and biochemical parameters—including age, sex, renal function categories, HbA1c, potassium, CRP, albumin, hemoglobin, and several concomitant medications—were not independently associated with delirium during this interval.

Collectively, these findings indicate that the lower delirium risk associated with GLP-1RA exposure persisted after adjustment for clinically relevant covariates. The concomitant association with statin therapy and the differential signal across LDL cholesterol strata further suggests that lipid-related or vascular-metabolic factors may contribute to post-AKD delirium risk, although these exploratory associations should not be interpreted as defining an optimal LDL target. As a sensitivity assessment for potential competing mortality, death occurred in 113 of 3,275 patients (3.5%) in the GLP-1RA group and 123 of 3,277 patients (3.8%) in the SGLT2i group. Mortality did not differ significantly between treatment groups (risk difference, −0.003; 95% CI, −0.012 to 0.006; P = 0.510; risk ratio, 0.919; 95% CI, 0.715–1.181).

### Early neuropsychiatric outcomes during 90–180 days

Analysis of the early post-AKD period revealed a different pattern (**Figure 4**). During days 90–180, the GLP-1RA group had a higher risk of depression relative to the SGLT2i group (HR, 1.84; 95% CI, 1.07–3.15; *P* = 0.023), corresponding to a lower early risk of depression among SGLT2i users.

**Figure 4:**
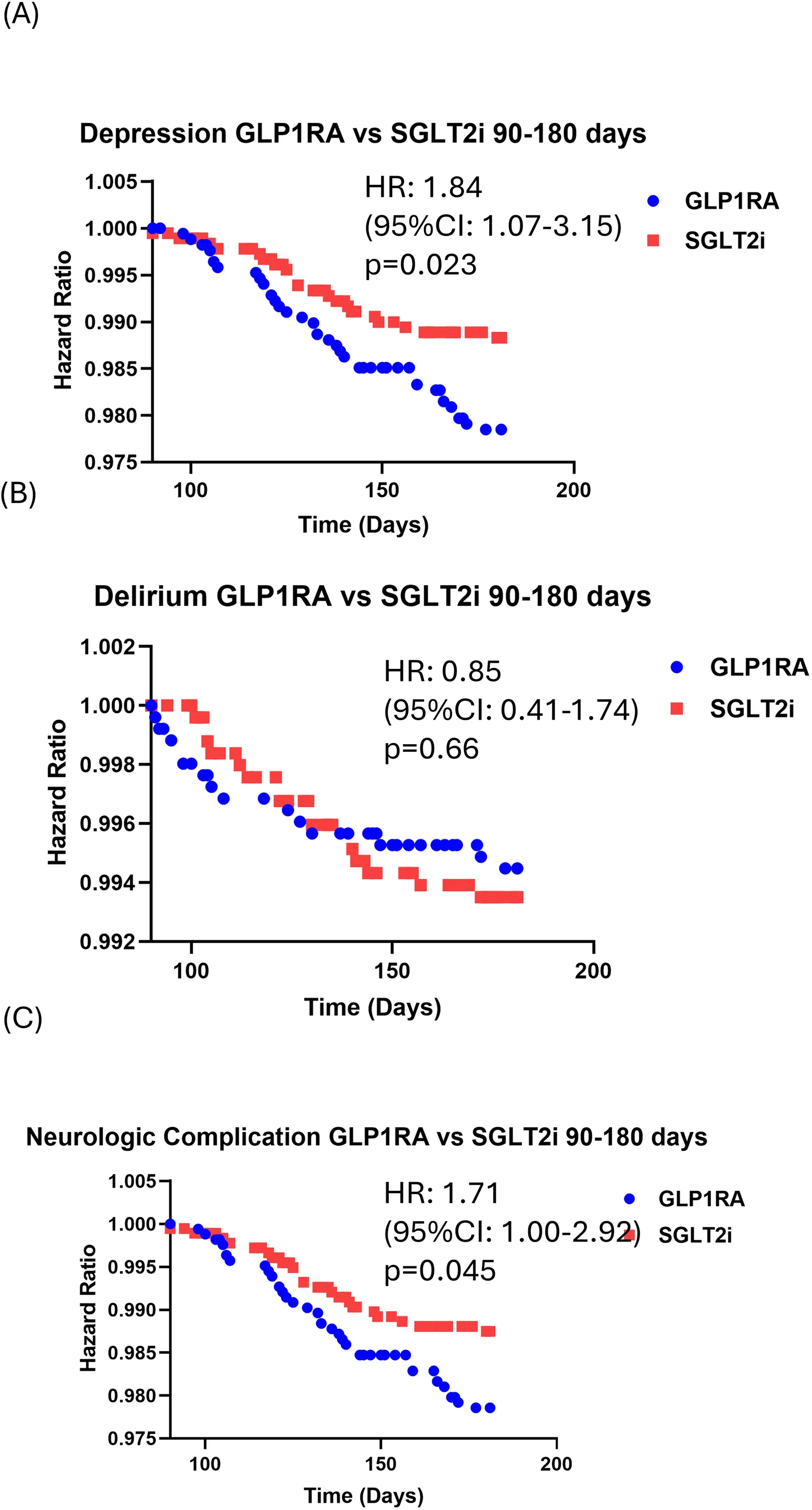
Early neurological outcomes associated with GLP-1RA versus SGLT2i use during the 90–180-day follow-up period. Time-to-event analyses comparing GLP-1RA and SGLT2i users during the early 90–180-day post-discharge period for **(A)** depression, **(B)** incident delirium, and **(C)** composite neurological complications. Compared with SGLT2i use, GLP-1RA use was associated with a higher risk of depression (HR, 1.84; 95% CI, 1.07–3.15; *P* = 0.023) and composite neurological complications (HR, 1.71; 95% CI, 1.00–2.92; *P* = 0.045), whereas no significant difference was observed for delirium (HR, 0.85; 95% CI, 0.41–1.74; *P* = 0.66). These findings indicate a comparatively favorable early neurological profile with SGLT2i, particularly for depression. CI, confidence interval; GLP-1RA, glucagon-like peptide-1 receptor agonist; HR, hazard ratio; SGLT2i, sodium–glucose cotransporter-2 inhibitor.

A similar early pattern was observed for the composite neurological outcome, with an HR of 1.71 (95% CI, 1.00–2.92; *P* = 0.045) for GLP-1RA versus SGLT2i. In contrast, no significant difference in incident delirium was detected during this early interval (HR, 0.85; 95% CI, 0.41–1.74; *P* = 0.660).

These findings suggest that the relative neurological associations of the two drug classes differed according to both outcome phenotype and follow-up period. Specifically, the early post-AKD period was characterized by a favorable association of SGLT2i exposure with depression and the composite neurological outcome, whereas a significant difference in delirium had not yet emerged.

### Extended neuropsychiatric outcomes during 90–730 days

Extended follow-up further demonstrated the temporal heterogeneity of these associations (**Figure 5**). The early difference in depression was no longer evident over the 90–730-day period (HR, 0.937; 95% CI, 0.748–1.175; *P* = 0.577). Similarly, there was no significant difference in the composite neurological outcome (HR, 0.91; 95% CI, 0.73–1.13; *P* = 0.416).

**Figure 5:**
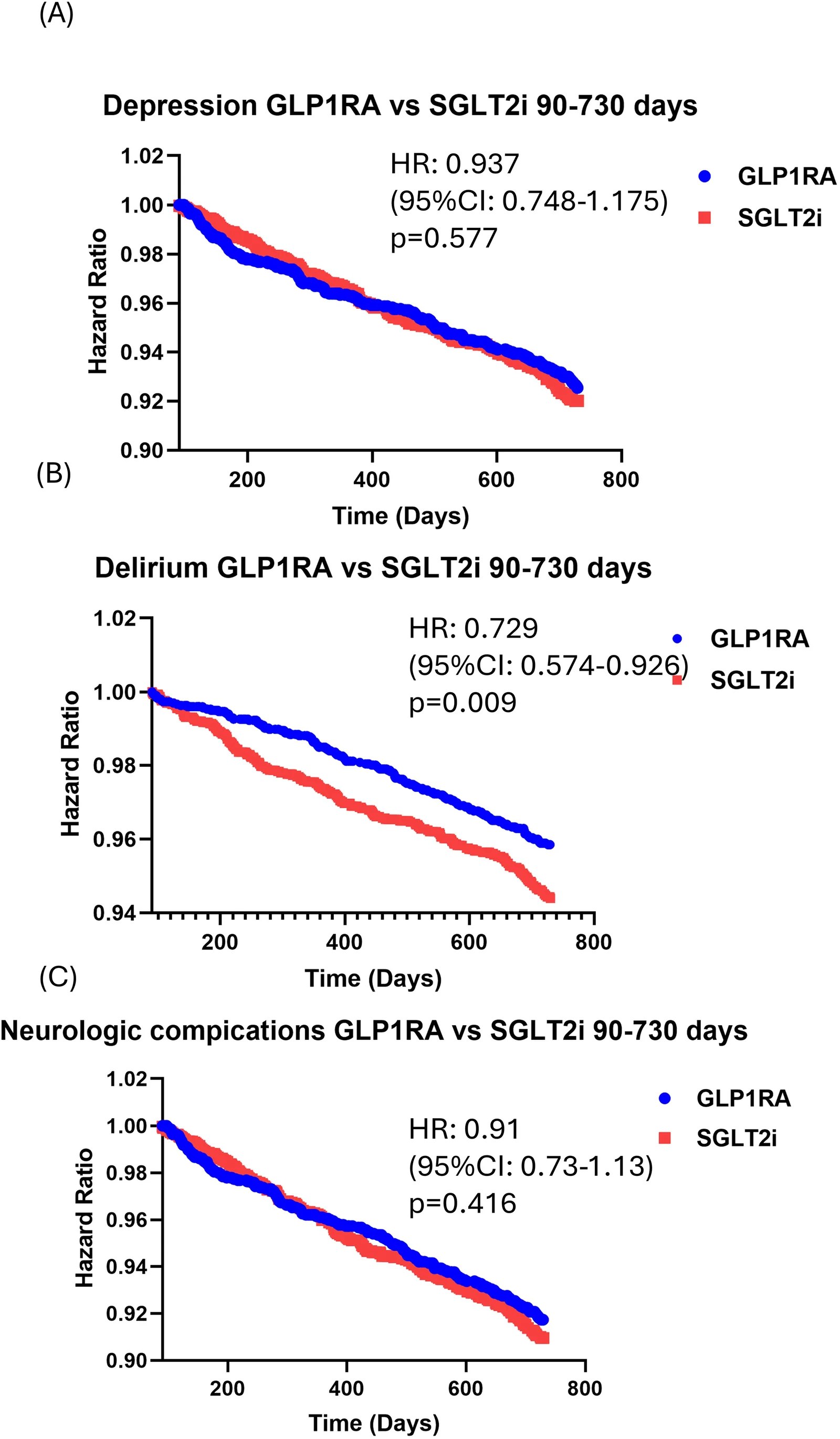
Extended neurological outcomes associated with GLP-1RA versus SGLT2i use during the 90–730-day follow-up period. Time-to-event analyses comparing GLP-1RA and SGLT2i users during the extended 90–730-day follow-up period for **(A)** depression, **(B)** incident delirium, and **(C)** composite neurological complications. The early difference in depression was no longer observed during extended follow-up (HR, 0.937; 95% CI, 0.748–1.175; *P* = 0.577). In contrast, GLP-1RA use remained associated with a significantly lower risk of incident delirium (HR, 0.729; 95% CI, 0.574–0.926; *P* = 0.009). No significant difference was observed for composite neurological complications (HR, 0.91; 95% CI, 0.73–1.13; *P* = 0.416). These findings support outcome-specific and temporally distinct associations between the two glucose-lowering therapies.

In contrast, the association between GLP-1RA exposure and lower delirium risk persisted during extended follow-up. GLP-1RA users had a significantly lower risk of incident delirium than SGLT2i users during the 90–730-day period (HR, 0.729; 95% CI, 0.574–0.926; *P* = 0.009).

Taken together, the analyses across the three follow-up windows revealed a distinct temporal and outcome-specific pattern. SGLT2i exposure was associated with a lower risk of depression during the early 90–180-day post-AKD period, but this association attenuated during longer follow-up. Conversely, the difference in delirium was not apparent during the early period but became evident during the 90–365-day primary analysis and remained significant through extended follow-up. Neither treatment demonstrated a consistent advantage for the overall composite neurological outcome across longer observation periods.

## Discussion

The first major finding of the present study was that GLP-1 receptor agonist (GLP-1RA) use was associated with a lower risk of incident delirium than SGLT2 inhibitor use during the 90–365-day period following dialysis-requiring acute kidney disease (AKD). This observation is clinically relevant because direct evidence linking GLP-1RA therapy specifically to delirium prevention remains limited, although an expanding body of evidence supports broader neurological effects of GLP-1 receptor activation. Several biologically plausible mechanisms may underlie the potential neuroprotective effects of GLP-1RAs. Experimental studies have shown that GLP-1RA treatment may attenuate neuroinflammation by modulating microglial activation and promoting a shift from pro-inflammatory toward anti-inflammatory microglial phenotypes (1, 2). In patients with type 2 diabetes, GLP-1RA therapy has also been associated with reduced oxidative stress and inflammatory signaling, together with improvements in mitochondrial membrane potential and respiratory function (3). Beyond these anti-inflammatory and mitochondrial effects, liraglutide has been shown to preserve cerebral glucose metabolism and improve blood–brain glucose transfer in patients with Alzheimer’s disease, suggesting a potential role in maintaining cerebral metabolic homeostasis (4, 5).

The apparent specificity of this association for delirium, rather than depression or the broader composite neurological outcome, may be particularly relevant to the underlying pathophysiology of delirium. Delirium represents an acute brain dysfunction that is highly sensitive to systemic inflammatory insults and has been mechanistically linked to neuroinflammation, microglial activation, endothelial dysfunction, blood–brain barrier disruption, oxidative stress, and impaired cerebral energy metabolism (6, 7). Such mechanistic convergence may be especially relevant after severe AKD, in which persistent systemic inflammatory, metabolic, and vascular disturbances may increase susceptibility to subsequent acute brain dysfunction.

In contrast, depression is a more heterogeneous and multifactorial neuropsychiatric disorder involving not only inflammatory pathways but also monoaminergic and glutamatergic neurotransmission, hypothalamic–pituitary–adrenal axis dysregulation, neurotrophic signaling, synaptic plasticity, and psychosocial factors (8). GLP-1RA-mediated inflammatory and metabolic modulation may not translate into a consistent antidepressant effect. Likewise, combining biologically distinct neuropsychiatric outcomes may dilute a delirium-specific association. Importantly, emerging human evidence supports an association between GLP-1RA exposure and reduced neurocognitive vulnerability. A population-based longitudinal study of 109,778 patients with type 2 diabetes reported a lower incidence of dementia among GLP-1RA users than nonusers (adjusted HR 0.90, 95% CI 0.83–0.97 (9)). Similarly, a large target-trial emulation of more than one million patients with type 2 diabetes found that semaglutide use was associated with a lower risk of first-time Alzheimer disease diagnosis than several comparator glucose-lowering therapies (10)]. These findings do not establish GLP-1RA as a treatment for delirium, but they provide clinical plausibility for our observation that GLP-1RA exposure may be associated with reduced neurological vulnerability during recovery from severe AKD. Notably, the treatment difference in delirium was not evident during the early 90–180-day period but emerged during the primary 90–365-day analysis and remained evident during extended follow-up. This temporal pattern raises the possibility that GLP-1RA-associated neurological effects may become more apparent during post-acute recovery rather than immediately after AKD. Nevertheless, given the observational nature of our study, these findings should be interpreted as temporal and outcome-specific associations rather than evidence of a direct causal effect of GLP-1RA therapy on delirium prevention.

The additional time-stratified analyses also highlight the influence of residual covariate imbalance on the observed associations. In the crude analyses, between-group differences in depression and overall neurological complications were primarily observed during the early 90–180-day interval, whereas these associations became more consistent across intervals after adjustment for age, sex, ischemic heart disease, diuretic use, and beta-blocker use. In contrast, delirium remained nonsignificantly different between treatment groups across all three non-overlapping intervals after adjustment. The differences between the crude and adjusted estimates suggest that residual imbalance after propensity score matching may have influenced some of the observed secondary neuropsychiatric associations. Given the multiple outcome- and interval-specific comparisons and the absence of formal multiplicity adjustment or treatment-by-time interaction testing, these findings should be considered exploratory and hypothesis-generating rather than confirmatory evidence of differential or time-dependent treatment effects.

A second finding was that statin use was independently associated with a lower risk of incident delirium, whereas LDL-C levels of 100–129.9 mg/dL showed an exploratory association with a higher delirium hazard. Importantly, the LDL-C ranges in our analysis were evaluated as separate indicator variables rather than as mutually exclusive categories using a single LDL-C stratum as the reference. These observations should not necessarily be interpreted as manifestations of the same mechanism. Rather, they may reflect complementary lipid-related and lipid-independent pathways influencing neurological vulnerability after AKD. The association confined to an intermediate LDL-C range is particularly intriguing because the relationship between cholesterol and neurological outcomes may be nonlinear. In patients with type 2 diabetes, Zhang et al. demonstrated an inverted U-shaped association between LDL-C and cognitive performance, with an estimated inflection point of 2.686 mmol/L, approximately 104 mg/dL (11). Although that study evaluated cognitive function rather than delirium, it supports the concept that neurological outcomes may not follow a simple linear relationship with LDL-C concentration. In contrast, the association between statin exposure and delirium has more direct clinical support. A propensity score-matched study of 110,090 older adults with type 2 diabetes found a lower incidence of delirium among statin users than matched nonusers (12). Earlier evidence in critically ill patients similarly suggested a reduced risk of delirium during statin administration and proposed attenuation of systemic inflammation as a potential mechanism (13). Beyond LDL-C lowering, statins exert several pleiotropic effects, including attenuation of inflammation and oxidative stress and improvement of endothelial function (14). These effects may also extend to the neurovascular unit, where statins have been proposed to preserve blood–brain barrier integrity, reduce endothelial inflammatory responses, and modulate neuroinflammation (15, 16). Such mechanisms are potentially relevant to delirium, a condition strongly linked to systemic inflammation, endothelial dysfunction, and disruption of the blood–brain barrier. Notably, previous clinical evidence in critically ill patients has suggested an association between statin exposure and a reduced risk of delirium (13). Therefore, our findings may indicate that lipid status and statin exposure provide complementary information regarding susceptibility to post-AKD delirium. Nevertheless, the LDL-C 100–129.9 mg/dL finding should be regarded as exploratory and should not be interpreted as establishing this range as an optimal LDL-C target for delirium prevention.

The third major observation was a favorable association between SGLT2 inhibitor use and depression during the earlier post-AKD period, which was no longer evident with extended follow-up. Emerging real-world studies have consistently associated SGLT2 inhibitor use with a lower incidence of depression in patients with type 2 diabetes, although these studies did not specifically characterize an early, time-limited effect (17, 18). SGLT2 inhibitors exert rapid metabolic and hemodynamic effects after treatment initiation, including early improvements in glycemic dynamics (19), providing a potential biological basis for a stronger association during the vulnerable early recovery phase after AKD. Glycemic status may also influence these associations. HbA1c reflects chronic glycemic exposure and has recently been linked to broader metabolic and microvascular vulnerability (20, 21). In our cohort, HbA1c remained slightly higher in GLP-1RA users after matching (9.0% vs. 8.6%; SMD = 0.139). Because longitudinal HbA1c changes were not assessed, whether differential glycemic improvement contributed to the observed neuropsychiatric associations remains uncertain.More broadly, recent real-world analyses of glucose-lowering therapies have demonstrated that neuropsychiatric associations may differ between early and later follow-up periods, supporting the relevance of landmark-based temporal assessment (22). Therefore, the disappearance of the depression signal during extended follow-up in our study should be interpreted as a novel, hypothesis-generating temporal pattern rather than confirmation of an established time-limited antidepressant effect of SGLT2 inhibition. Taken together, our findings suggest outcome-specific and temporally heterogeneous neuropsychiatric associations after AKD, with an early favorable depression profile associated with SGLT2i use and a later, sustained reduction in incident delirium associated with GLP-1RA use.

## Limitations

Several limitations should be acknowledged. First, this retrospective EHR-based study remains susceptible to residual and unmeasured confounding despite propensity score matching and multivariable adjustment. Complete balance was not achieved for several clinically relevant characteristics, including BMI and cardiovascular comorbidities. Residual imbalance may reflect confounding by indication, as ADA guidelines recommend GLP-1RAs and SGLT2 inhibitors according to distinct metabolic, cardiovascular, and kidney profiles (23, 24); therefore, causal inference remains limited. Second, death was treated as a censoring event in the conventional Kaplan–Meier and Cox analyses rather than explicitly modeled as a competing event. However, mortality was relatively low and similar between the GLP-1RA and SGLT2i groups (3.5% vs. 3.8%; P = 0.510), reducing concern that differential mortality substantially accounted for the observed between-group associations. Nevertheless, residual competing-risk bias cannot be excluded, and future studies with patient-level data should confirm these findings using competing-risk methods. Third, delirium and depression were identified using diagnostic codes rather than standardized prospective assessments. Claims-based algorithms for delirium have high specificity but relatively low sensitivity, potentially resulting in underrecognition and outcome misclassification (25). Medication exposure recorded in EHRs also does not ensure adherence or continued treatment, and treatment switching or discontinuation during follow-up may introduce exposure misclassification (26, 27). Furthermore, incomplete laboratory data warrant cautious interpretation of the LDL-C and statin findings, particularly the LDL-C 100–129.9 mg/dL signal, which should be considered hypothesis-generating. Finally, the different associations across the 90–180-, 90–365-, and 90–730-day windows do not establish a formal treatment-by-time interaction and require prospective validation. Generalizability beyond patients with T2DM and dialysis-requiring AKD may also be limited.

## Conclusion

In this retrospective cohort study of patients with type 2 diabetes following dialysis-requiring acute kidney disease, GLP-1 receptor agonist and SGLT2 inhibitor use were associated with outcome-specific neuropsychiatric profiles. Although the primary analysis suggested a lower risk of incident delirium with GLP-1RA use, this association was not consistently observed in adjusted analyses across non-overlapping follow-up intervals. In contrast, adjusted interval-specific analyses showed a higher risk of depression and overall neurological complications with GLP-1RA compared with SGLT2i use. Statin use was independently associated with a lower risk of delirium, suggesting a potential contribution of vascular-metabolic and inflammatory pathways to post-AKD neuropsychiatric vulnerability. Given the observational design, residual confounding, and multiple interval-specific comparisons, these findings should be considered exploratory and hypothesis-generating. Prospective studies are needed to validate these associations and determine whether treatment effects truly vary over time.

## Data Availability

The data analyzed in this study is subject to the following licenses/restrictions: The underlying TriNetX patient-level data are not publicly available because of data use agreements and patient privacy restrictions. Access is restricted to authorized users through participating institutions. Requests to access these datasets should be directed to https://trinetx.com/#amy-jo.
