# Supplementary material for: Distinct temporal neuropsychiatric outcomes associated with GLP-1 receptor agonists versus SGLT2 inhibitors after acute kidney disease in type 2 diabetes
Source: Front Endocrinol (Lausanne). 2026 Oct 27;17:1963944. doi: 10.3389/fendo.2026.1963944 (PMC13747840; doi:10.3389/fendo.2026.1963944)
Supplement: Supplementary file 1 [file Table1.docx]

**Supplementary Table S1. Coding definitions for cohort construction, exposures, acute kidney disease, baseline covariates, and study outcomes**

Coding definitions were organized according to the TriNetX cohort exports used for the study. ICD-10-CM, CPT, HCPCS, SNOMED CT, ICD-9-CM, ATC, and LOINC concepts are shown where applicable. For laboratory variables, the listed LOINC codes represent the standardized laboratory concepts used for reproducible reporting; the exact local source code may vary across contributing healthcare organizations.

| **Domain** | **Parameter / definition** | **Coding system** | **Code(s)** | **Operational definition / notes** |
| --- | --- | --- | --- | --- |
| Cohort construction | Age | Demographic | Age 18–80 years | Adults aged 18–80 years at the most recent qualifying occurrence. |
| Cohort construction | Type 2 diabetes mellitus | ICD-10-CM | E11 | Required diabetes diagnosis in the study cohort definition. |
| Exposure | GLP-1 receptor agonists (GLP-1RA) | ATC | A10BJ | Glucagon-like peptide-1 analogues. GLP-1RA cohort required exposure to A10BJ and excluded A10BK. |
| Exposure | SGLT2 inhibitors (SGLT2i) | ATC | A10BK | Sodium-glucose cotransporter-2 inhibitors. SGLT2i cohort required exposure to A10BK and excluded A10BJ. |
| Exposure | Representative GLP-1RA agents | ATC drug level | A10BJ01 exenatide; A10BJ02 liraglutide; A10BJ03 lixisenatide; A10BJ05 dulaglutide; A10BJ06 semaglutide; A10BJ07 beinaglutide; A10BJ08 albiglutide; A10BJ09 tirzepatide* | Class exposure was defined at ATC A10BJ level in TriNetX; individual agents are provided to clarify class membership. *ATC classification may evolve over time. |
| Exposure | Representative SGLT2i agents | ATC drug level | A10BK01 dapagliflozin; A10BK02 canagliflozin; A10BK03 empagliflozin; A10BK04 ertugliflozin; A10BK05 ipragliflozin; A10BK06 sotagliflozin | Class exposure was defined at ATC A10BK level in TriNetX; individual agents are provided to clarify class membership. |
| AKD / index hospitalization | Hospital discharge, >30 min | CPT | 99239 | Hospital inpatient or observation discharge day management; >30 minutes. |
| AKD / index hospitalization | Hospital discharge, ≤30 min | CPT | 99238 | Hospital inpatient or observation discharge day management; ≤30 minutes. |
| AKD / index hospitalization | Observation discharge | CPT | 99217 | Observation care discharge-day management. |
| AKD / index hospitalization | Hospital inpatient/observation discharge services | TriNetX/CPT concept | 1013682 | TriNetX grouped discharge-services concept. |
| AKD / renal replacement therapy | Hemodialysis, repeated evaluation | CPT | 90937 | Hemodialysis requiring repeated evaluation(s), with or without revision of dialysis prescription. |
| AKD / renal replacement therapy | Hemodialysis, single evaluation | CPT | 90935 | Hemodialysis with single physician/qualified professional evaluation. |
| AKD / renal replacement therapy | Non-hemodialysis dialysis/CRRT, repeated evaluation | CPT | 90947 | Dialysis other than hemodialysis, including peritoneal dialysis, hemofiltration, or other continuous renal replacement therapy, with repeated evaluations. |
| AKD / renal replacement therapy | Non-hemodialysis dialysis/CRRT, single evaluation | CPT | 90945 | Dialysis other than hemodialysis, including peritoneal dialysis/hemofiltration/CRRT, with single evaluation. |
| AKD / renal replacement therapy | Hemodialysis procedures | TriNetX/CPT concept | 1012752 | Grouped hemodialysis-procedure concept. |
| AKD / renal replacement therapy | Dialysis services and procedures | TriNetX/CPT concept | 1012740 | Grouped dialysis-services/procedures concept. |
| AKD / renal replacement therapy | Hemodialysis | SNOMED CT | 302497006 | Hemodialysis procedure. |
| AKD / renal replacement therapy | Hemodialysis | ICD-9-CM | 39.95 | Hemodialysis procedure. |
| AKD / renal replacement therapy | Dialysis dependence | ICD-10-CM | Z99.2 | Dependence on renal dialysis. |
| AKD / renal replacement therapy | Short-term dialysis catheter | HCPCS | C1752 | Catheter, hemodialysis/peritoneal, short-term. |
| AKD / associated diagnosis | Fluid, electrolyte, and acid-base disorder | ICD-10-CM | E87 | Other disorders of fluid, electrolyte, and acid-base balance; included in the exported renal/dialysis concept set. |
| AKD / temporal rule | Hospital dialysis relative to discharge | Management rule | — | A qualifying hospital-dialysis event occurred within 3 months before through 1 day after the discharge event in the exported cohort definition. |
| AKD / exclusion at hospitalization | Delirium / altered consciousness exclusions | ICD-10-CM | F05; R41.82; R41.0; R40.0 | Sensitive pre-index exclusion for delirium due to a known physiological condition, altered mental status, disorientation, and/or somnolence during the qualifying hospitalization according to the query export. |
| Baseline – demographics | Age at index | Demographic | Age at index | Continuous variable. |
| Baseline – demographics | Sex | Demographic | Male / Female | Sex recorded in TriNetX demographic data. |
| Baseline – comorbidity | Diabetes mellitus | ICD-10-CM | E08–E13 (study cohort primarily E11) | Diabetes mellitus; cohort construction specifically required type 2 diabetes mellitus (E11). |
| Baseline – comorbidity | Overweight/obesity | ICD-10-CM | E66 | Overweight and obesity. |
| Baseline – comorbidity | Hypertension | ICD-10-CM | I10–I16 | Hypertensive diseases. |
| Baseline – comorbidity | Ischemic heart disease | ICD-10-CM | I20–I25 | Ischemic heart diseases. |
| Baseline – comorbidity | Cerebrovascular disease | ICD-10-CM | I60–I69 | Cerebrovascular diseases. |
| Baseline – comorbidity | COPD | ICD-10-CM | J44 | Other chronic obstructive pulmonary disease. |
| Baseline – comorbidity | Hyperlipidemia | ICD-10-CM | E78 | Disorders of lipoprotein metabolism and other lipidemias. |
| Baseline – laboratory | BMI | LOINC | 39156-5 | Body mass index, kg/m². |
| Baseline – laboratory | eGFR (CKD-EPI 2021) | LOINC | 98979-8 | Glomerular filtration rate/1.73 m² calculated by creatinine-based CKD-EPI 2021 formula. Categories reported as ≥60, 30–59, and <30 mL/min/1.73 m². |
| Baseline – laboratory | Urine albumin-to-creatinine ratio (UACR) | LOINC | 14959-1 | Microalbumin/creatinine mass ratio in urine, mg/g. |
| Baseline – laboratory | Hemoglobin | LOINC | 718-7 | Hemoglobin concentration in blood, g/dL. |
| Baseline – laboratory | Total cholesterol | LOINC | 2093-3 | Cholesterol, total, serum/plasma, mg/dL. |
| Baseline – laboratory | LDL cholesterol | LOINC | 13457-7 | LDL cholesterol, calculated or reported standardized concept, mg/dL. |
| Baseline – laboratory | HDL cholesterol | LOINC | 2085-9 | HDL cholesterol, serum/plasma, mg/dL. |
| Baseline – laboratory | Triglycerides | LOINC | 2571-8 | Triglycerides, serum/plasma, mg/dL. |
| Baseline – laboratory | HbA1c | LOINC | 4548-4 | Hemoglobin A1c/Hemoglobin.total in blood, %. |
| Baseline – laboratory | Potassium | LOINC | 2823-3 | Potassium, serum/plasma, mmol/L. |
| Baseline – medication | DPP-4 inhibitors | ATC | A10BH | Dipeptidyl peptidase-4 inhibitors. |
| Baseline – medication | Biguanides | ATC | A10BA | Biguanides, principally metformin. |
| Baseline – medication | RAAS inhibitors | ATC | C09 | Agents acting on the renin-angiotensin system. |
| Baseline – medication | Beta-blockers | ATC | C07 | Beta-blocking agents. |
| Baseline – medication | Diuretics | ATC | C03 | Diuretics. |
| Baseline – medication | Calcium-channel blockers | ATC | C08 | Calcium-channel blockers. |
| Baseline – medication | Statins | ATC | C10AA | HMG-CoA reductase inhibitors. |
| Baseline – medication | Benzodiazepine derivatives | ATC | N05BA | Benzodiazepine derivatives. |
| Baseline – medication | Antipsychotics | ATC | N05A | Antipsychotics. |
| Outcome | Delirium | ICD-10-CM / SNOMED CT / RxNorm | F05; R40.1; R40.0; R41.82; SNOMED CT 386255004; RxNorm 51272; and the additional delirium-related diagnosis concepts listed in the original TriNetX outcome export | Primary neurological outcome. Delirium was defined using the complete TriNetX outcome concept set shown in the original query export, including delirium due to a known physiological condition, stupor, somnolence, altered mental status, delirium-management procedures, quetiapine, and the listed substance-related and dementia-related delirium diagnosis concepts. The outcome was therefore not restricted to ICD-10-CM F05. |
| Outcome | Dementia – unspecified | ICD-10-CM | F03 and descendants | Unspecified dementia, including severity/behavioral-disturbance subcodes captured by the TriNetX hierarchy. |
| Outcome | Dementia in other diseases | ICD-10-CM | F02 and descendants | Dementia in other diseases classified elsewhere. |
| Outcome | Vascular dementia | ICD-10-CM | F01 and descendants | Vascular dementia. |
| Outcome | Alzheimer disease | ICD-10-CM | G30 and descendants | Alzheimer disease. |
| Outcome | Amnestic disorder | ICD-10-CM | F04 | Amnestic disorder due to known physiological condition; included in the composite cognitive outcome definition. |
| Outcome | Mild cognitive impairment (MCI) | ICD-10-CM | G31.84; F09 | Mild cognitive impairment of uncertain/unknown etiology and unspecified mental disorder due to known physiological condition. |
| Outcome | Depression | ICD-10-CM | F32; F33; F06.31; F43.21 | Depressive episode; recurrent major depressive disorder; mood disorder due to known physiological condition with depressive features; adjustment disorder with depressed mood. |
| Outcome | Composite neurocognitive outcome | ICD-10-CM | F01; F02; F03; F04; F05; F09; F32; F33; F06.31; F43.21; G30; G31.84 | Composite of dementia, delirium, depression, and MCI as defined in the TriNetX outcome analysis. |
| Outcome analysis rule | Incident outcome handling | TriNetX analytic setting | Exclude patients with outcome prior to time window | Patients with the specified outcome before the start of the analysis window were excluded from the corresponding risk and Kaplan–Meier analyses. |

**Legend:** This supplementary table provides reproducible coding definitions for cohort construction, treatment exposure, the operational AKD/hospital dialysis phenotype, baseline variables reported in Table 1, and neurological outcomes. The original TriNetX export defined GLP-1RA and SGLT2i exposure using ATC class codes A10BJ and A10BK, respectively. The exported AKD cohort was operationalized through a hospitalization/discharge plus renal replacement therapy/dialysis concept set and temporal relationship; therefore, the table reports the actual electronic phenotype rather than substituting a purely laboratory-based KDIGO AKD definition.

**Abbreviations:** AKD, acute kidney disease; ATC, Anatomical Therapeutic Chemical classification; BMI, body mass index; CKD-EPI, Chronic Kidney Disease Epidemiology Collaboration; COPD, chronic obstructive pulmonary disease; CPT, Current Procedural Terminology; CRRT, continuous renal replacement therapy; DPP-4, dipeptidyl peptidase-4; eGFR, estimated glomerular filtration rate; GLP-1RA, glucagon-like peptide-1 receptor agonist; HbA1c, glycated hemoglobin A1c; HCPCS, Healthcare Common Procedure Coding System; HDL, high-density lipoprotein; ICD-10-CM, International Classification of Diseases, Tenth Revision, Clinical Modification; LDL, low-density lipoprotein; LOINC, Logical Observation Identifiers Names and Codes; MCI, mild cognitive impairment; RAAS, renin-angiotensin-aldosterone system; SGLT2i, sodium-glucose cotransporter-2 inhibitor; SNOMED CT, Systematized Nomenclature of Medicine Clinical Terms; UACR, urine albumin-to-creatinine ratio.

**Technical note:** TriNetX uses standardized terminologies mapped from heterogeneous source electronic health records. ATC and diagnosis/procedure codes above reflect the exported study definitions. Laboratory LOINC codes are supplied as standardized reporting mappings; local laboratory identifiers may differ among participating healthcare organizations.
