# Supplementary material for: Distinct temporal neuropsychiatric outcomes associated with GLP-1 receptor agonists versus SGLT2 inhibitors after acute kidney disease in type 2 diabetes
Source: Front Endocrinol (Lausanne). 2026 Oct 27;17:1963944. doi: 10.3389/fendo.2026.1963944 (PMC13747840; doi:10.3389/fendo.2026.1963944)
Supplement: Supplementary file 2 [file Table2.docx]

**Supplementary Table 2. Crude Kaplan-Meier analyses across non-overlapping follow-up windows**

| **Outcome** | **Time window** | **GLP-1RA Events/N** | **SGLT2i Events/N** | **Crude KM HR (95% CI)** | **Log-rank P** |
| --- | --- | --- | --- | --- | --- |
| Depression | 90-180 d | 47/2,451 | 32/2,640 | 1.572 (1.003-2.463) | 0.0465 |
|  | 181-365 d | 50/2,324 | 49/2,491 | 1.081 (0.729-1.603) | 0.6992 |
|  | 366-730 d | 83/2,272 | 100/2,433 | 0.865 (0.647-1.158) | 0.3293 |
| Delirium | 90-180 d | 14/2,576 | 16/2,535 | 0.852 (0.416-1.745) | 0.6605 |
|  | 181-365 d | 37/3,508 | 48/3,455 | 0.747 (0.486-1.146) | 0.1801 |
|  | 366-730 d | 88/3,462 | 97/3,401 | 0.868 (0.650-1.158) | 0.3354 |
| Overall neurological complications | 90-180 d | 48/2,405 | 33/2,596 | 1.559 (1.001-2.428) | 0.0478 |
|  | 181-365 d | 55/2,275 | 57/2,439 | 1.022 (0.705-1.480) | 0.9091 |
|  | 366-730 d | 93/2,218 | 105/2,379 | 0.925 (0.699-1.222) | 0.5821 |

Abbreviations: CI, confidence interval; GLP-1RA, glucagon-like peptide-1 receptor agonist; HR, hazard ratio; KM, Kaplan-Meier; SGLT2i, sodium-glucose cotransporter-2 inhibitor. HRs compare GLP-1RA with SGLT2i; HR >1 indicates a higher hazard in the GLP-1RA cohort, whereas HR <1 indicates a lower hazard in the GLP-1RA cohort. Patients with the corresponding outcome before the beginning of each time window were excluded from the risk set. P values were derived from log-rank tests. Analyses across alternative follow-up windows were exploratory, and P values were nominal without adjustment for multiple comparisons.
