# Supplementary material for: Distinct temporal neuropsychiatric outcomes associated with GLP-1 receptor agonists versus SGLT2 inhibitors after acute kidney disease in type 2 diabetes
Source: Front Endocrinol (Lausanne). 2026 Oct 27;17:1963944. doi: 10.3389/fendo.2026.1963944 (PMC13747840; doi:10.3389/fendo.2026.1963944)
Supplement: Supplementary file 3 [file Table3.docx]

**Supplementary Table 3. Multivariable-adjusted Cox analyses across non-overlapping follow-up windows**

| **Outcome** | **Follow-up window (days)** | **Adjusted HR** | **95% CI** | **P value** |
| --- | --- | --- | --- | --- |
| Overall neurological complications | 90-180 | 1.259 | 1.120-1.416 | 0.0001 |
|  | 181-365 | 1.269 | 1.151-1.399 | <0.0001 |
|  | 366-730 | 1.207 | 1.109-1.314 | <0.0001 |
| Depression | 90-180 | 1.319 | 1.170-1.488 | <0.0001 |
|  | 181-365 | 1.337 | 1.208-1.481 | <0.0001 |
|  | 366-730 | 1.256 | 1.150-1.372 | <0.0001 |
| Delirium | 90-180 | 0.778 | 0.528-1.148 | 0.2067 |
|  | 181-365 | 0.814 | 0.609-1.089 | 0.1660 |
|  | 366-730 | 0.926 | 0.735-1.168 | 0.5180 |

Adjusted for age, sex, ischemic heart disease, diuretic use, and beta-blocker use. Hazard ratios compare GLP-1RA with SGLT2i; therefore, HR >1 indicates a higher hazard in the GLP-1RA cohort, whereas HR <1 indicates a lower hazard in the GLP-1RA cohort. Analyses across alternative follow-up windows were exploratory, and P values were nominal without adjustment for multiple comparisons. Abbreviations: CI, confidence interval; GLP-1RA, glucagon-like peptide-1 receptor agonist; HR, hazard ratio; SGLT2i, sodium-glucose cotransporter-2 inhibitor.
