# Supplementary material for: Distinct temporal neuropsychiatric outcomes associated with GLP-1 receptor agonists versus SGLT2 inhibitors after acute kidney disease in type 2 diabetes
Source: Front Endocrinol (Lausanne). 2026 Oct 27;17:1963944. doi: 10.3389/fendo.2026.1963944 (PMC13747840; doi:10.3389/fendo.2026.1963944)
Supplement: Supplementary file 4 [file Table4.docx]

**Supplementary Table S4. Agent-specific sensitivity analysis comparing empagliflozin and semaglutide for neurological and neuropsychiatric outcomes**

| **Outcome** | **Empagliflozin, events/N** | **Semaglutide, events/N** | **HR (95% CI)** | **Log-rank P value** |
| --- | --- | --- | --- | --- |
| Depression | 41/1,226 | 44/1,142 | 0.880 (0.575–1.347) | 0.5570 |
| Delirium | 35/1,743 | 23/1,734 | 1.556 (0.920–2.634) | 0.0966 |
| Overall neurological complications | 53/1,208 | 46/1,122 | 1.091 (0.735–1.620) | 0.6645 |

**Footnote.** Empagliflozin and semaglutide were selected because they were the most frequently represented individual agents within the SGLT2i and GLP-1RA cohorts, respectively. Relative to the corresponding pre-matching class-level cohort sizes, empagliflozin accounted for approximately 83.7% (4,971/5,937) of SGLT2i users and semaglutide for approximately 53.3% (2,039/3,822) of GLP-1RA users. After 1:1 propensity score matching, 1,951 patients were included in each agent-specific cohort. Outcome-specific denominators differ because patients with the corresponding outcome before the assessment window were excluded. HRs represent empagliflozin relative to semaglutide. These analyses were exploratory.
